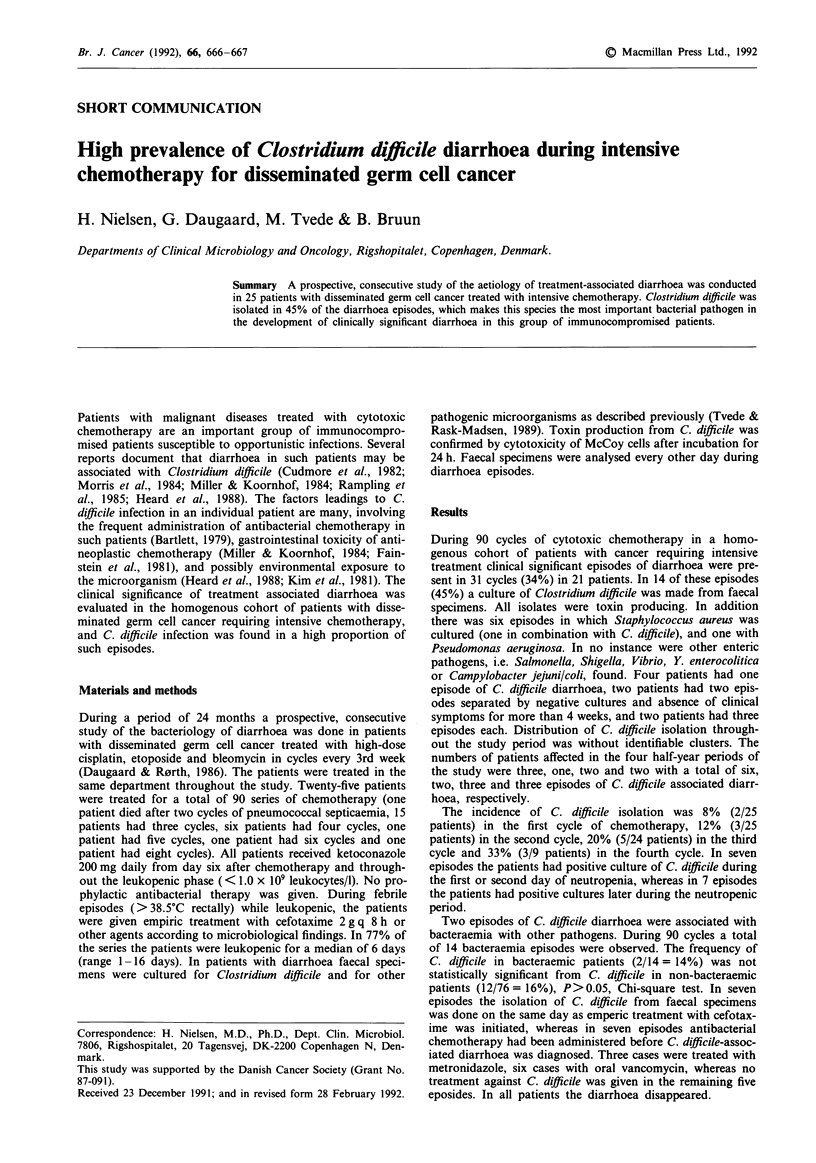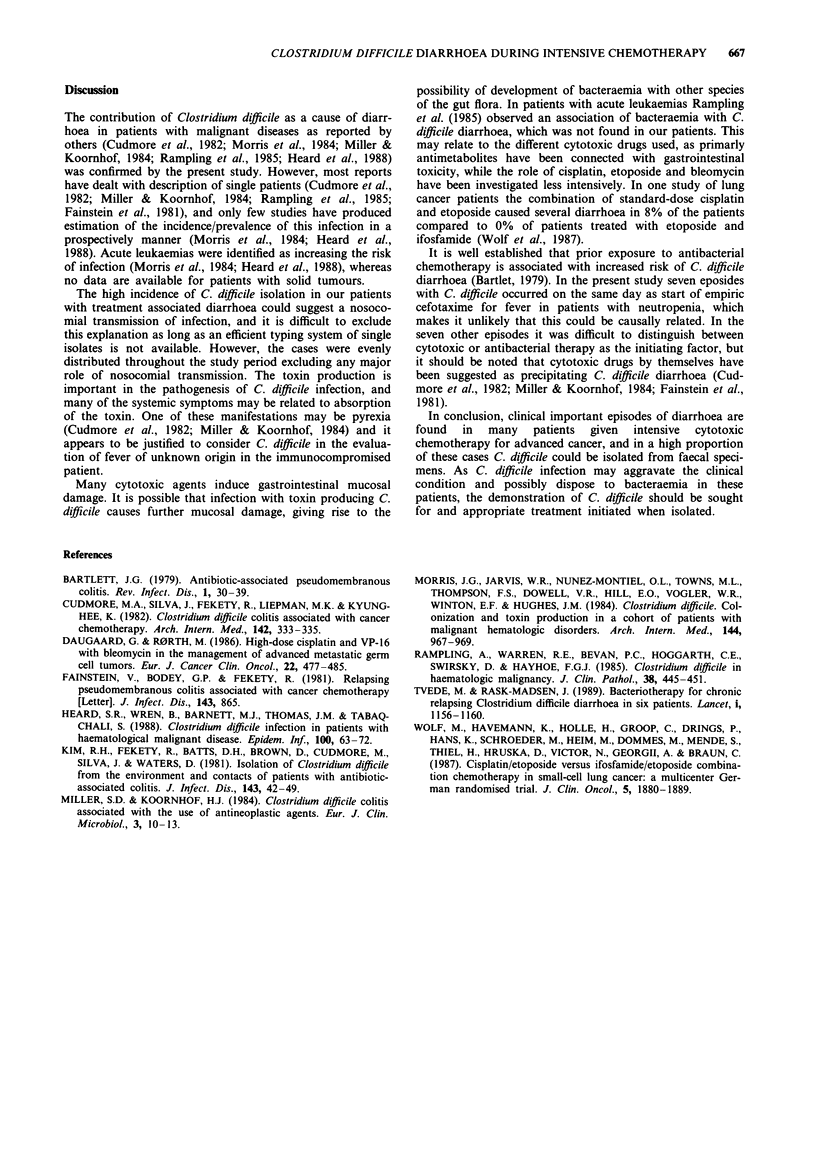# High prevalence of Clostridium difficile diarrhoea during intensive chemotherapy for disseminated germ cell cancer.

**DOI:** 10.1038/bjc.1992.334

**Published:** 1992-10

**Authors:** H. Nielsen, G. Daugaard, M. Tvede, B. Bruun

**Affiliations:** Department of Clinical Microbiology, Rigshopitalet, Copenhagen, Denmark.

## Abstract

A prospective, consecutive study of the aetiology of treatment-associated diarrhoea was conducted in 25 patients with disseminated germ cell cancer treated with intensive chemotherapy. Clostridium difficile was isolated in 45% of the diarrhoea episodes, which makes this species the most important bacterial pathogen in the development of clinically significant diarrhoea in this group of immunocompromised patients.


Br. J. Cancer (1992), 66, 666-667 © Macmillan Press Ltd., 1992

SHORT COMMUNICATION

### High prevalence of *Clostridium difficile* diarrhoea during intensive chemotherapy for disseminated germ cell cancer

H. Nielsen, G. Daugaard, M. Tvede & B. Bruun

*Departments of Clinical Microbiology and Oncology, Rigshopitalet, Copenhagen, Denmark.*

**Summary** A prospective, consecutive study of the aetiology of treatment-associated diarrhoea was conducted in 25 patients with disseminated germ cell cancer treated with intensive chemotherapy. *Clostridium difficile* was isolated in 45% of the diarrhoea episodes, which makes this species the most important bacterial pathogen in the development of clinically significant diarrhoea in this group of immunocompromised patients.

Patients with malignant diseases treated with cytotoxic chemotherapy are an important group of immunocompromised patients susceptible to opportunistic infections. Several reports document that diarrhoea in such patients may be associated with *Clostridium difficile* (Cudmore *et al.*, 1982; Morris *et al.*, 1984; Miller & Koornhof, 1984; Rampling *et al.*, 1985; Heard *et al.*, 1988). The factors leadings to *C. difficile* infection in an individual patient are many, involving the frequent administration of antibacterial chemotherapy in such patients (Bartlett, 1979), gastrointestinal toxicity of antineoplastic chemotherapy (Miller & Koornhof, 1984; Fainstein *et al.*, 1981), and possibly environmental exposure to the microorganism (Heard *et al.*, 1988; Kim *et al.*, 1981). The clinical significance of treatment associated diarrhoea was evaluated in the homogenous cohort of patients with disseminated germ cell cancer requiring intensive chemotherapy, and *C. difficile* infection was found in a high proportion of such episodes.

### Materials and methods

During a period of 24 months a prospective, consecutive study of the bacteriology of diarrhoea was done in patients with disseminated germ cell cancer treated with high-dose cisplatin, etoposide and bleomycin in cycles every 3rd week (Daugaard & Rørth, 1986). The patients were treated in the same department throughout the study. Twenty-five patients were treated for a total of 90 series of chemotherapy (one patient died after two cycles of pneumococcal septicaemia, 15 patients had three cycles, six patients had four cycles, one patient had five cycles, one patient had six cycles and one patient had eight cycles). All patients received ketoconazole 200 mg daily from day six after chemotherapy and throughout the leukopenic phase ($< 1.0 \times 10^9$ leukocytes/l). No prophylactic antibacterial therapy was given. During febrile episodes ($> 38.5$°C rectally) while leukopenic, the patients were given empiric treatment with cefotaxime 2 g q 8 h or other agents according to microbiological findings. In 77% of the series the patients were leukopenic for a median of 6 days (range 1-16 days). In patients with diarrhoea faecal specimens were cultured for *Clostridium difficile* and for other pathogenic microorganisms as described previously (Tvede & Rask-Madsen, 1989). Toxin production from *C. difficile* was confirmed by cytotoxicity of McCoy cells after incubation for 24 h. Faecal specimens were analysed every other day during diarrhoea episodes.

### Results

During 90 cycles of cytotoxic chemotherapy in a homogenous cohort of patients with cancer requiring intensive treatment clinical significant episodes of diarrhoea were present in 31 cycles (34%) in 21 patients. In 14 of these episodes (45%) a culture of *Clostridium difficile* was made from faecal specimens. All isolates were toxin producing. In addition there was six episodes in which *Staphylococcus aureus* was cultured (one in combination with *C. difficile*), and one with *Pseudomonas aeruginosa*. In no instance were other enteric pathogens, i.e. *Salmonella, Shigella, Vibrio, Y. enterocolitica* or *Campylobacter jejuni/coli*, found. Four patients had one episode of *C. difficile* diarrhoea, two patients had two episodes separated by negative cultures and absence of clinical symptoms for more than 4 weeks, and two patients had three episodes each. Distribution of *C. difficile* isolation throughout the study period was without identifiable clusters. The numbers of patients affected in the four half-year periods of the study were three, one, two and two with a total of six, two, three and three episodes of *C. difficile* associated diarrhoea, respectively.

The incidence of *C. difficile* isolation was 8% (2/25 patients) in the first cycle of chemotherapy, 12% (3/25 patients) in the second cycle, 20% (5/24 patients) in the third cycle and 33% (3/9 patients) in the fourth cycle. In seven episodes the patients had positive culture of *C. difficile* during the first or second day of neutropenia, whereas in 7 episodes the patients had positive cultures later during the neutropenic period.

Two episodes of *C. difficile* diarrhoea were associated with bacteraemia with other pathogens. During 90 cycles a total of 14 bacteraemia episodes were observed. The frequency of *C. difficile* in bacteraemic patients (2/14 = 14%) was not statistically significant from *C. difficile* in non-bacteraemic patients (12/76 = 16%), $P > 0.05$, Chi-square test. In seven episodes the isolation of *C. difficile* from faecal specimens was done on the same day as emperic treatment with cefotaxime was initiated, whereas in seven episodes antibacterial chemotherapy had been administered before *C. difficile*-associated diarrhoea was diagnosed. Three cases were treated with metronidazole, six cases with oral vancomycin, whereas no treatment against *C. difficile* was given in the remaining five eposides. In all patients the diarrhoea disappeared.

Correspondence: H. Nielsen, M.D., Ph.D., Dept. Clin. Microbiol. 7806, Rigshospitalet, 20 Tagensvej, DK-2200 Copenhagen N, Denmark.
This study was supported by the Danish Cancer Society (Grant No. 87-091).
Received 23 December 1991; and in revised form 28 February 1992.

### Discussion

The contribution of *Clostridium difficile* as a cause of diarrhoea in patients with malignant diseases as reported by others (Cudmore *et al.*, 1982; Morris *et al.*, 1984; Miller & Koornhof, 1984; Rampling *et al.*, 1985; Heard *et al.*, 1988) was confirmed by the present study. However, most reports have dealt with description of single patients (Cudmore *et al.*, 1982; Miller & Koornhof, 1984; Rampling *et al.*, 1985; Fainstein *et al.*, 1981), and only few studies have produced estimation of the incidence/prevalence of this infection in a prospectively manner (Morris *et al.*, 1984; Heard *et al.*, 1988). Acute leukaemias were identified as increasing the risk of infection (Morris *et al.*, 1984; Heard *et al.*, 1988), whereas no data are available for patients with solid tumours.

The high incidence of *C. difficile* isolation in our patients with treatment associated diarrhoea could suggest a nosocomial transmission of infection, and it is difficult to exclude this explanation as long as an efficient typing system of single isolates is not available. However, the cases were evenly distributed throughout the study period excluding any major role of nosocomial transmission. The toxin production is important in the pathogenesis of *C. difficile* infection, and many of the systemic symptoms may be related to absorption of the toxin. One of these manifestations may be pyrexia (Cudmore *et al.*, 1982; Miller & Koornhof, 1984) and it appears to be justified to consider *C. difficile* in the evaluation of fever of unknown origin in the immunocompromised patient.

Many cytotoxic agents induce gastrointestinal mucosal damage. It is possible that infection with toxin producing *C. difficile* causes further mucosal damage, giving rise to the possibility of development of bacteraemia with other species of the gut flora. In patients with acute leukaemias Rampling *et al.* (1985) observed an association of bacteraemia with *C. difficile* diarrhoea, which was not found in our patients. This may relate to the different cytotoxic drugs used, as primarly antimetabolites have been connected with gastrointestinal toxicity, while the role of cisplatin, etoposide and bleomycin have been investigated less intensively. In one study of lung cancer patients the combination of standard-dose cisplatin and etoposide caused several diarrhoea in 8% of the patients compared to 0% of patients treated with etoposide and ifosfamide (Wolf *et al.*, 1987).

It is well established that prior exposure to antibacterial chemotherapy is associated with increased risk of *C. difficile* diarrhoea (Bartlet, 1979). In the present study seven eposides with *C. difficile* occurred on the same day as start of empiric cefotaxime for fever in patients with neutropenia, which makes it unlikely that this could be causally related. In the seven other episodes it was difficult to distinguish between cytotoxic or antibacterial therapy as the initiating factor, but it should be noted that cytotoxic drugs by themselves have been suggested as precipitating *C. difficile* diarrhoea (Cudmore *et al.*, 1982; Miller & Koornhof, 1984; Fainstein *et al.*, 1981).

In conclusion, clinical important episodes of diarrhoea are found in many patients given intensive cytotoxic chemotherapy for advanced cancer, and in a high proportion of these cases *C. difficile* could be isolated from faecal specimens. As *C. difficile* infection may aggravate the clinical condition and possibly dispose to bacteraemia in these patients, the demonstration of *C. difficile* should be sought for and appropriate treatment initiated when isolated.

### References

BARTLETT, J.G. (1979). Antibiotic-associated pseudomembranous colitis. *Rev. Infect. Dis.*, **1**, 30–39.

CUDMORE, M.A., SILVA, J., FEKETY, R., LIEPMAN, M.K. & KYUNG-HEE, K. (1982). *Clostridium difficile* colitis associated with cancer chemotherapy. *Arch. Intern. Med.*, **142**, 333–335.

DAUGAARD, G. & RØRTH, M. (1986). High-dose cisplatin and VP-16 with bleomycin in the management of advanced metastatic germ cell tumors. *Eur. J. Cancer Clin. Oncol.*, **22**, 477–485.

FAINSTEIN, V., BODEY, G.P. & FEKETY, R. (1981). Relapsing pseudomembranous colitis associated with cancer chemotherapy [Letter]. *J. Infect. Dis.*, **143**, 865.

HEARD, S.R., WREN, B., BARNETT, M.J., THOMAS, J.M. & TABAQCHALI, S. (1988). *Clostridium difficile* infection in patients with haematological malignant disease. *Epidem. Inf.*, **100**, 63–72.

KIM, R.H., FEKETY, R., BATTS, D.H., BROWN, D., CUDMORE, M., SILVA, J. & WATERS, D. (1981). Isolation of *Clostridium difficile* from the environment and contacts of patients with antibiotic-associated colitis. *J. Infect. Dis.*, **143**, 42–49.

MILLER, S.D. & KOORNHOF, H.J. (1984). *Clostridium difficile* colitis associated with the use of antineoplastic agents. *Eur. J. Clin. Microbiol.*, **3**, 10–13.

MORRIS, J.G., JARVIS, W.R., NUNEZ-MONTIEL, O.L., TOWNS, M.L., THOMPSON, F.S., DOWELL, V.R., HILL, E.O., VOGLER, W.R., WINTON, E.F. & HUGHES, J.M. (1984). *Clostridium difficile*. Colonization and toxin production in a cohort of patients with malignant hematologic disorders. *Arch. Intern. Med.*, **144**, 967–969.

RAMPLING, A., WARREN, R.E., BEVAN, P.C., HOGGARTH, C.E., SWIRSKY, D. & HAYHOE, F.G.J. (1985). *Clostridium difficile* in haematologic malignancy. *J. Clin. Pathol.*, **38**, 445–451.

TVEDE, M. & RASK-MADSEN, J. (1989). Bacteriotherapy for chronic relapsing Clostridium difficile diarrhoea in six patients. *Lancet*, **i**, 1156–1160.

WOLF, M., HAVEMANN, K., HOLLE, H., GROOP, C., DRINGS, P., HANS, K., SCHROEDER, M., HEIM, M., DOMMES, M., MENDE, S., THIEL, H., HRUSKA, D., VICTOR, N., GEORGII, A. & BRAUN, C. (1987). Cisplatin/etoposide versus ifosfamide/etoposide combination chemotherapy in small-cell lung cancer: a multicenter German randomised trial. *J. Clin. Oncol.*, **5**, 1880–1889.